# Urban sustainability in the global south

**DOI:** 10.1038/s41598-026-39080-8

**Published:** 2026-02-13

**Authors:** Linchuan Yang, Aya Hagishima

**Affiliations:** 1 Department of Urban and Rural Planning, School of Architecture, Southwest Jiaotong University, Chengdu, China; 2 Faculty of Engineering Sciences, Kyushu University, Kasuga City, Fukuoka, Japan

**Keywords:** Psychology and behaviour, Civil engineering

## Abstract

Urbanization in the Global South is accelerating amid a confluence of ecological, social, and developmental pressures that diverge considerably from those experienced in the Global North. Recent advances in spatial analysis, environmental modeling, and related fields are reshaping scholarly understandings of these multifaceted challenges and the targeted policy responses needed to address them. The *Urban Sustainability in the Global South* Collection, published in *Scientific Reports*, compiles interdisciplinary research contributions that elucidate ongoing urban transformations and provide evidence-based insights to inform pathways toward inclusive, safe, resilient, and sustainable urban futures, aligned with Sustainable Development Goal 11 (SDG 11).

The trajectory of global urbanization underscores the pressing need to address urban sustainability, particularly as a substantial proportion of current and future urban growth is predicted to occur in the Global South^1^. The discourse on urban sustainability has, however, long exhibited an implicit bias toward cities in the Global North, which have frequently been positioned as models for emulation^2^. This perspective insufficiently acknowledges the distinct and complex conditions that shape urban development in the Global South, including rapid demographic transitions, informality, fiscal and institutional constraints, colonial planning legacies, and heightened vulnerability to climate-related risks^3,4^. Therefore, achieving sustainable urban development in this context necessitates a decisive shift in perspective.

Instead of framing the discourse on how cities of the Global South can simply “catch up,” we argue for an empirically grounded reframing that starts from local biophysical and socioeconomic conditions and leads to context-specific solutions^2^. This shift is also consistent with evidence that climate-related urban risks are intensifying and are shaped by local exposure, vulnerability, and governance capacity, particularly in rapidly growing cities.

This Collection, *Urban Sustainability in the Global South*, was dedicated to supporting and amplifying research aligned with Sustainable Development Goal 11 (SDG 11), *Sustainable Cities and Communities*. It aimed to bring together a diverse range of contributions that not only document the multifaceted challenges confronting cities of the Global South but also shed light on pathways toward inclusive, safe, resilient, and sustainable urban futures.

Nine papers are included in this Collection. They cover diverse countries, including but not limited to China, Pakistan, Egypt, Iran, India, and South Africa. Methodologically, they employ advanced data-driven approaches, including geographic information systems, remote sensing technologies, and state-of-the-art econometrics and statistical techniques. Together, these papers provide nuanced and empirically grounded insights into various critical areas, such as climate vulnerability and social equity in green infrastructure. They can be broadly categorized into two groups: urban green spaces (3 papers) and urban sustainability and resilience (6 papers).

Urban green spaces not only play a pivotal role in shaping human activity behaviors and promoting healthy lifestyles but also serve as essential infrastructure for urban sustainability^5–7^. Kifayatullah et al.^8^ employed GIS-based spatial analysis to examine the spatial distribution, typology, and functionality of urban green spaces in Islamabad, Pakistan, revealing pronounced spatial inequities. For example, wealthier areas possessed larger and better-maintained green spaces. The authors argue that these disparities compromise both ecological integrity and social cohesion and thus advocate data-driven planning to advance the equitable provision of green space. Complementing this supply-side perspective, Mohamed and Kronenberg^9^ analyzed users’ perceptions of park accessibility and attractiveness in Cairo, Egypt, by mining social media data (specifically, online reviews). They revealed that user-generated content provides urban planners and practitioners with nuanced insights that can inform evidence-based planning and management decisions. Maleknia and Svobodova^10^ investigated the behavioral determinants of Iranian female high-school students’ intentions to conserve urban forests through an extended theory of planned behavior. The authors found that attitudes, perceived behavioral control, environmental awareness, and social responsibility emerged as significant predictors, whereas subjective norms did not. Consequently, they emphasized the pivotal role of cultivating environmental responsibility and practical skills to foster youth engagement in urban forest conservation.

As emphasized in SDG 11, urban sustainability and resilience are of paramount importance to cities worldwide. Hzami et al.^11^ evaluated the vulnerability of coastal energy infrastructure in Doha, Qatar, under diverse sea-level rise scenarios. Their projections indicated that by 2100, nearly 60% of the city’s land area and 39% of its residential power units will be at risk of inundation. Their analysis underscores the urgent necessity for integrated adaptation strategies aimed at safeguarding coastal infrastructure and enhancing energy resilience. In the context of heat-related risks, Ramachandra et al.^12^ investigated the linkages between urban heat islands and landscape morphology in Bangalore, India. Their findings revealed that landscapes covered with vegetation and water bodies serve as critical heat sinks, playing a vital role in mitigating urban heat. Shifting the focus from climatic hazards to social development, Avtar et al.^13^ examined the impact of built-up population density on human well-being in Delhi, India. They found that while moderate density can improve access to services, excessive density exacerbates infrastructure pressure, reduces green space availability, and intensifies resource stress. Consequently, they proposed context-specific (place-varying) urban planning strategies to address these challenges. Zhang et al.^14^ explored the environmental implications of China’s super urban agglomeration strategy. They demonstrated that industrial aggregation generally has an inverted U-shaped effect on industrial pollution, though this effect varies across urban agglomerations at different stages of development. They argued that context-specific industrial agglomeration policies and cross-regional environmental governance are crucial for balancing economic efficiency with ecological sustainability. Bai and Shen^15^ analyzed the influence of the digital economy on sustainable urban development across 30 underdeveloped cities in northwest China. Their findings underscore the critical importance of developing differentiated digital strategies to foster inclusive and sustainable urban transformation processes. Du Plessis et al.^16^ explored the feasibility of integrating co-creation approaches into conventional landscape design practices, while carefully considering the diverse interests of relevant stakeholders.

Collectively, the papers in this Collection underscore the inherent complexity of urban sustainability in the Global South, while demonstrating that localized, data-driven, and context-specific approaches are indispensable. They offer timely and evidence-based guidance for policymakers and practitioners striving to build inclusive, safe, resilient, and sustainable urban futures. Future research and practice can be advanced along several key directions: (1) developing innovative methodologies and data governance systems to enhance the monitoring and holistic understanding of urban transformation processes across the Global South; (2) strengthening data-informed planning frameworks that safeguard equitable access to healthy and livable environments for all residents; (3) exploring urban–rural linkages to strengthen ecological resilience, consolidate food security, and promote regional sustainability; and (4) deepening locally grounded and inclusive approaches that integrate diverse cultural norms, institutional arrangements, and multi-level governance perspectives.
